# Large Angiomatous Nasal Polyp Presents With Epistaxis Imitating Juvenile Nasopharyngeal Angiofibroma

**DOI:** 10.7759/cureus.45239

**Published:** 2023-09-14

**Authors:** John M Coggins, Brian P Quinlan, Madelyn L Schmidt, Ran A Wang, Charles A Hughes

**Affiliations:** 1 Department of Otolaryngology, University of Texas Medical Branch, Galveston, USA

**Keywords:** maxillary sinus, epistaxis, nasopharyngeal angiofibroma, sinonasal polyp, angiomatous nasal polyp (anp)

## Abstract

An angiomatous nasal polyp is a rare subtype of sinonasal polyp that is commonly found in the middle meatus and characterized by the presence of blood vessels within polyp tissue. It is a benign lesion but is prone to misdiagnosis as a malignant tumor because it typically grows larger and is more vascular than other types of polyps. In this report, a 16-year-old male with no significant past medical history presents with a six-month history of epistaxis and progressive nasal obstruction. Examination of the oral cavity showed a centrally located soft palate mass. CT maxillofacial with contrast showed a hypervascular 3.4 x 4.7 x 6.1 cm mass in the nasal cavity extending through the nasal choanae and down to the level of the tongue. MRI showed a heterogenous polypoid mass originating from the left middle meatus vs. nasal cavity, with characteristics favoring an aggressive tumor. The patient was taken for interventional radiology (IR) embolization and nasal endoscopy. Biopsy showed the left nasal mass contained granulation tissue and the palatal mass consisted of necrotic tissue. He was taken for second-stage endoscopic sinus surgery with plans for extensive reconstruction if necessary. Extensive polyposis was found without gross evidence of an aggressive tumor. The anterior polyposis was debulked and the polyp was cut at its root to allow for removal of the nasopharyngeal/oropharyngeal portion through the mouth. He was able to be discharged on the same day and his postoperative recovery was uncomplicated. Angiomatous nasal polyps are uncommon, share features of aggressive tumors on imaging, and require angiography and biopsy to safely rule out malignancy. Endoscopic surgical resection typically results in good outcomes and low recurrence rates.

## Introduction

Angiomatous nasal polyps (ANPs) are a rare subtype of sinonasal polyps characterized by vascular proliferation within the polyp. They comprise 4%-5% of all sinonasal polyps and likely develop from secondary changes of sinonasal polyps as they grow and develop vascular compromise with subsequent neovascularization [1,2]. The majority of ANPs originate in the maxillary sinus and extend into the choana and nasopharynx [3]. Common clinical presentations include a history of progressive nasal obstruction, epistaxis, anosmia, and rhinorrhea [4]. CT imaging will show an expansile, heterogenous mass. Pathology will show expanded angiogenesis, eosinophils, and atypical stromal cells [5]. ANPs are prone to be misdiagnosed as malignant tumors or juvenile nasopharyngeal angiofibroma (JNA) due to their similar appearance. A careful review of imaging and pathology findings is necessary to determine the correct diagnosis. Transnasal endoscopic removal is the treatment of choice and has low rates of recurrence [6].

## Case presentation

**Figure 1:**
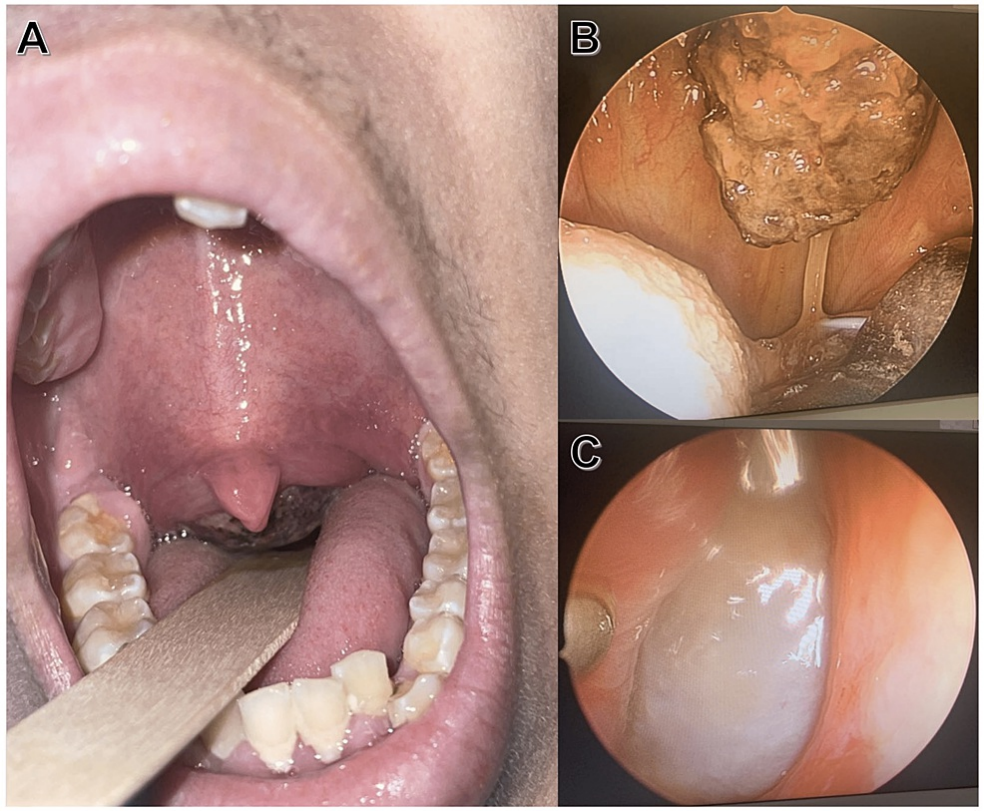
Oral exam and nasopharyngoscopy (A) Necrotic nasopharyngeal mass extending into the oropharynx posterior to the uvula. Images B and C were taken during nasopharyngoscopy. (B) Necrotic nasopharyngeal mass. (C) A right nasopharyngeal polyp.

A 16-year-old male with no significant past medical history presents with a six-month history of epistaxis and nasal obstruction. Three months ago, the bleeding became severe enough that the patient was withdrawn from school. He was originally seen at another institution and given antibiotics for foul-smelling nasal discharge. He also reports an eight lb. weight loss over the last month. On exam, the patient was noted to be solely mouth-breathing due to nasal obstruction. His nose was normal externally without septal deviation, with bilateral polyposis and abundant drainage noted. The oral cavity exhibited a centrally located soft palate mass which pressed the soft palate inferiorly, pointing the uvula anteriorly (Figures 1A-1C). The oropharynx displayed no erythema or lesions, and the head and neck had no palpable lymphadenopathy.

**Figure 2:**
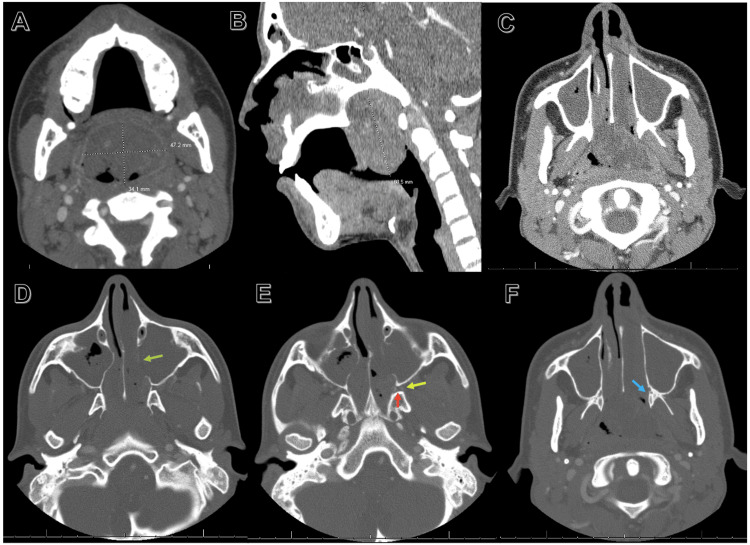
CT maxillofacial/mandible with contrast (A-C) A large mass centered at the nasopharynx measuring 3.4 x 4.7 x 6.1 cm. Internal serpiginous foci of hyperdensity were noted within the mass resembling hypervascularity. The mass extends anteriorly through the nasal choanae and inferiorly to the level of the tongue, displacing the uvula anteriorly. There is a resultant narrowing of the airway at this level. (D) Mixed attenuation complex fluid filling the maxillary sinuses. The left maxillary sinus wall appears eroded (green arrow). (E) Pterygopalatine fossa (red arrow) and pterygomaxillary fissure (yellow arrow), which are unchanged in size when compared with the right side. (F) Medial pterygoid plate (blue arrow), which is unchanged when compared with the right side.

CT with contrast showed a hypervascular 3.4 x 4.7 x 6.1 cm mass that extended from the nasal cavity to the oropharynx (Figures 2A-2F). The left maxillary sinus demonstrated erosion of the medial wall, making it the likely site of origin. Posterior maxillary sinus bowing (Holman-Miller sign) was not noted and the pterygopalatine fossa and pterygomaxillary fissure were not widened, making JNA less likely. All of the paranasal sinuses demonstrated mixed attenuation accumulation of fluid except for the right frontal and right superior ethmoidal sinuses, likely reactive. Left-sided cervical lymphadenopathy was also noted.

**Figure 3:**
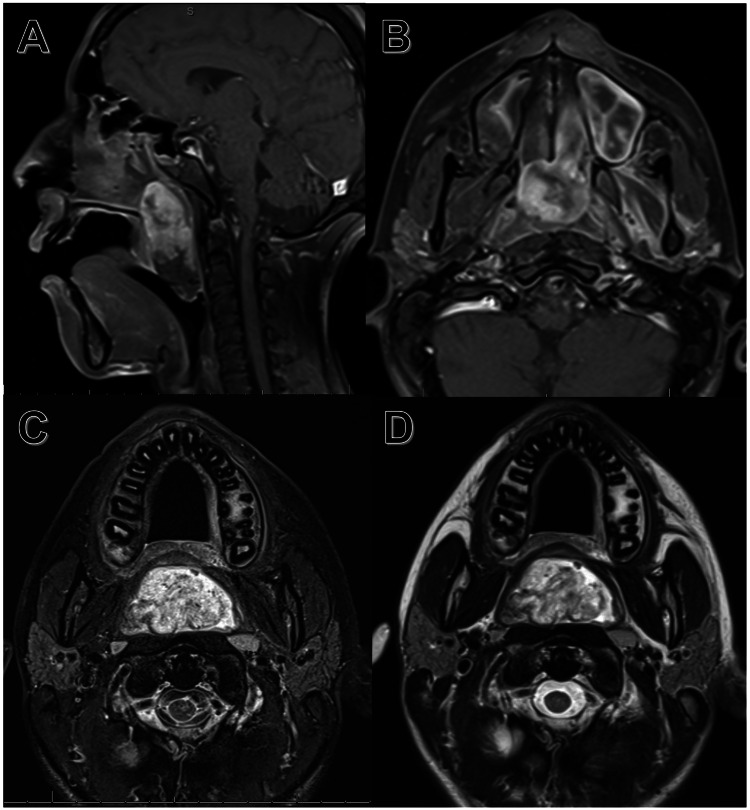
MRI face/paranasal sinuses with and without contrast (A) T1-weighted MRI image with contrast showing the nasopharyngeal polypoid mass. (B) T1-weighted MRI image with contrast showing extension into the left masticator space. (C) Short tau inversion recovery (STIR) MRI image without contrast showing a hyperintense mass. (D) T2-weighted MRI image without contrast showing a hyperintense mass.

MRI of the face and neck revealed a large, heterogeneously enhancing polypoid mass that appeared to arise from the left nasal mucosa or left maxillary sinus and extend into the nasopharynx and oropharynx (Figures 3A-3D). Abnormal signal enhancement involved the left pterygopalatine fossa, and the mass was noted to extend into the left mastication space. The left maxillary sinus medial wall appeared eroded, which may be secondary to vascular invasion or chronic inflammatory processes. Sinonasal carcinoma or rhabdomyosarcoma was favored based on the radiological findings.

**Figure 4:**
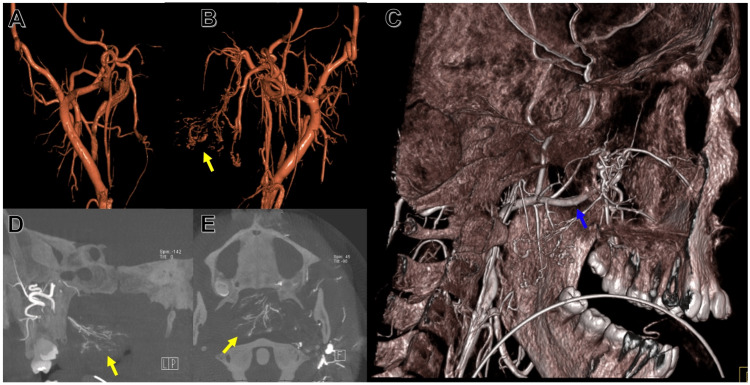
Cerebral angiogram (A) Angiography of the right external carotid artery with three-dimensional reconstruction from an anterior perspective. (B) Angiography of the left external carotid artery with three-dimensional reconstruction from an anterior perspective. Notable findings include hyperemic nasal mucosal and tumor blush (yellow arrow) from branches of the distal left internal maxillary artery. (C) Angiography of the left external carotid artery with a three-dimensional reconstruction of the skull. The internal maxillary artery (blue arrow) can be seen supplying the vasculature of the nasopharyngeal mass. (D) Angiography of the left external carotid artery from a posterolateral view with the tumor blush indicated by the yellow arrow. (E) Angiography of the left external carotid artery with an axial view and the tumor blush indicated by a yellow arrow.

An arteriogram performed under awake orotracheal intubation revealed hyperemic nasal mucosal and a tumor blush from branches of the distal left internal maxillary artery (IMAX) (Figures 4A-4E). The polyp was successfully embolized using polyvinylalcohol (PVA). While intubated, nasal endoscopy was also performed in the interventional radiology (IR) suite using a mobile setup. A zero-degree rigid endoscope was used to evaluate the nose, which showed diffuse polyposis in both nasal cavities, heavier on the left side. The scope could be passed to the nasopharynx on the right side, which showed a firm, brown mass. Two biopsies were obtained of the nasopharyngeal mass, which showed granulation tissue without malignancy and necrotic tissue with colonies of bacteria without malignancy. He was started on minocycline (100 mg daily) for two weeks prior to surgery for infection prophylaxis. One month later, the patient underwent endoscopic sinus surgery to debulk the polyposis and further assess the origin and features of the mass. A left maxillary antrostomy was also performed and polypoid tissue within the left maxillary sinus was debulked. The necrotic nasopharyngeal mass was biopsied and sent for frozen and permanent analyses. After frozen specimens were confirmed to be benign, the stalk of the nasopharyngeal mass (which was found at the middle meatus and posterior head of the inferior turbinate) was cut using a through-cut forceps, and the mass was pulled through the mouth. The final pathology of the nasopharyngeal mass revealed an angiomatous inflammatory nasal polyp with no malignancy identified. Seven weeks after surgery, the patient had recovered well and upon physical exam showed no signs of recurrence or synechia on nasal endoscopy.

## Discussion

ANPs are an uncommon nasopharyngeal soft tissue mass, most frequently diagnosed in children and young adults [7]. The polyps are often antrochoanal, originating in the maxillary sinus and extending through the medial meatus towards the choana. It is hypothesized that the polyp develops vascular compromise while growing due to compression by surrounding structures, most commonly the ostium exit site, posterior end of the inferior turbinate, choana, or nasopharynx [2]. The presentation, pathology, and radiology of ANP can often be confounded with other diagnoses, including JNA and malignant tumors. On CT imaging, ANP are typically non-enhancing soft tissue. On MRI, ANP typically does not show signal voids [8].

Biopsy can be used to distinguish malignant tumors from ANP, and angiography is generally required to distinguish JNA and ANP [8]. The paradoxical hypovascularity of ANP is caused by abnormal arborization resulting in racemose vasculature [9]. ANP vascularity may arise from any vessel in the nasal cavity, while JNA typically arises from arteries other than the anterior or posterior ethmoid. The distinction between these diagnoses is crucial as JNA poses a significant risk of bleeding and requires embolization before surgical resection, whereas ANP can be resected without prior embolization [8].

This report demonstrates the diagnostic approach to ANP and characteristic radiological findings, while also featuring several uncommon characteristics. First, MRI demonstrated erosion of the medial wall of the maxillary sinus, a feature which has only rarely occurred in benign lesions and is more typical of malignant tumors [10]. Also, this patient presented with recurrent epistaxis, which is an uncommon feature of ANP and initially led to suspicion of a JNA given his age and sex [11]. While both ANP and JNA typically occur in adolescence, JNA possesses a strong preference for males, which caused concern in this patient. Lastly, this patient’s ANP was significantly larger than is normally seen and was plainly visible upon oral exam; most ANPs do not extend significantly into the nasopharynx [8].

## Conclusions

ANPs are a rare variant of sinonasal polyps that are characterized by their distinctive histopathological features and findings on angiography. They typically present with symptoms of nasal obstruction, rhinorrhea, and anosmia, and are often misdiagnosed as other benign or malignant sinonasal tumors. This case of an ANP demonstrated several features atypical of the diagnosis, including a chief complaint of epistaxis, a large polyp extending into the oropharynx, and erosion of the medial wall of the maxillary sinus. The patient underwent successful excision of the nasal polyp endoscopically and recovered without complications. This report highlights the size to which ANP may grow and the possibility for ANP to present with epistaxis as the chief complaint. Providers should keep ANP on the differential for patients with similar presentations while cautiously evaluating for malignant tumors and JNA.
